# Tom Boys

**Published:** 1858-12

**Authors:** 


### TOM BOYS.

HOW we love the phrase! How it carries us back to the good old times when girls were not afraid to laugh out a whole heart at once, and never knew any thing of modern "propriety," sanctity before folks, satanity behind; angelic in the street, animal in the pantry, and in the study asinine!

"Tom Boys" is associated in our mind with Saleratus. Saleratus rises and helps to rise, so does a tom boy, for she is so full of romping and of fun, that with her joyous nature, and her unsuspicious abandon, she fires up every young heart around her, and makes the saddened faces of the old beam with the subdued but sweet smiles of the memories of Auld Lang Syne, when they too were young.

The first time we ever saw that now household word, "Saleratus," was when we were just beginning to take lessons in "Corderii," the first Latin primer. There was the picture of an angel broke loose. It was a young girl, with her long hair floating back in the breeze, an uncontrollable joyousness in her face, and withal, a most unsuspicious don't-care look about her; she was not on earth or in heaven, but between the two, in mid-air like, as if she had taken a spring, which was to end in a somerset, landing her right side up; and under this picture, was in large letters, Sal Eratus. Our first impulse was to "translate that." "Sal," we confidently believed, meant Sal, and "eratus," had something to do with erring, so we concluded that if Sal and erring were put together, it would make, in plain English "Erring Sal;" and that somebody's daughter, named Sal, would very probably, if she "cut up so," in the end, "put her foot in it," that is, "spoil the broth," or in other words, make a fool of herself, which means to take her pigs to a poor market, that is to say, would come out at the little end of the horn. Will any spirit about us vouchsafe an ability to express our idea in more courtly phrase, and better adapted to the modern market? For we ran back a moment to old times; and its association, its "aura," as Prof. Hall would say, so enveloped us, that we were "possessed" of old words, phrases, comparisons, old everything: specially did it bring to our mind, of how we went a moonshiny night to a prayer-meeting in the country, with Dr. Clelland's daughter, and how, when an essay was made to help her over the fence, with the tip end of a gloved finger, she exclaimed, "Oh, get out," and laying one hand on the top rail, she cleared the the panel at a bound! We felt mean for a whole year.

How sigh we for the times to come again, when for a girl to laugh out-right, to clear a fence, to reach the saddle at a bound, or row a river, or gallop alone to a neighbor's five miles and back, shall be considered nothing remarkable, its "symptom" being, an index to physical health, to joyous good nature, and, possession of high moral and physical abilities. How would a regiment of the true "tom boys" of olden times, quartered on Gotham, work a revolution for the better, in mind and morals, in physical elevation and mental power, whose influences for good would be felt for generations!

